# A miR-34a-guided, tRNA_i_^Met^-derived, piR_019752-like fragment (tRiMetF31) suppresses migration and angiogenesis of breast cancer cells via targeting PFKFB3

**DOI:** 10.1038/s41420-022-01054-w

**Published:** 2022-08-12

**Authors:** Bo Wang, Dongping Li, Yaroslav Ilnytskyy, Igor Kovalchuk, Olga Kovalchuk

**Affiliations:** Department of Biological Sciences, University of Lethbridge, Lethbridge, AB T1K 3M4 Canada

**Keywords:** Non-coding RNAs, Epigenetics, Breast cancer

## Abstract

Although we recently demonstrated that miR-34a directly targets tRNA_i_^Met^ precursors via Argonaute 2 (AGO2)-mediated cleavage, consequently attenuating the proliferation of breast cancer cells, whether tRNA_i_^Met^ fragments derived from this cleavage influence breast tumor angiogenesis remains unknown. Here, using small-RNA-Seq, we identified a tRNA_i_^Met^-derived, piR_019752-like 31-nt fragment tRiMetF31 in breast cancer cells expressing miR-34a. Bioinformatic analysis predicted 6-phosphofructo-2-kinase/fructose-2,6-bisphosphatase 3 (PFKFB3) as a potential target of tRiMrtF31, which was validated by luciferase assay. tRiMetF31 was downregulated, whereas PFKFB3 was overexpressed in cancer cell lines. Overexpression of tRiMetF31 profoundly inhibited the migration and angiogenesis of two breast cancer cell lines while slightly inducing apoptosis. Conversely, knockdown of tRiMetF31 restored PFKFB3-driven angiogenesis. miR-34a was downregulated, whereas tRNA_i_^Met^ and PFKFB3 were upregulated in breast cancer, and elevated PFKFB3 significantly correlated with metastasis. Our findings demonstrate that tRiMetF31 profoundly suppresses angiogenesis by silencing PFKFB3, presenting a novel target for therapeutic intervention in breast cancer.

## Introduction

Since microRNAs lin-4 and let-7 were first discovered in the developmental timing of *Caenorhabditis elegans* in 2001 [1–3], thousands of miRNA molecules have been identified in humans, and their functions have been extensively investigated. miRNAs are an abundant class of small non-coding RNAs (sncRNAs) that generally govern gene expression through either translational repression or mRNA degradation, which depends upon the complementarity of their sequences to 3’ untranslated regions (3’UTRs) of cognate mRNAs [4]. Although 3’UTRs are the most common region currently used in miRNA target prediction tools, miRNA targeting may also occur in 5’ untranslated regions (5’UTRs), open reading frames, and coding sequences [5–7]. To date, at least 60% of human protein-coding genes are conserved targets of miRNAs [8], reflecting the diverse functions of miRNA molecules in many biologic and pathologic processes. Because miRNAs can serve as oncogenes, tumor suppressors, or both [9–13], it comes as no surprise that dysregulated miRNAs profoundly contribute to tumorigenesis, angiogenesis, and metastasis [11, 12, 14–16].

Over 10 years ago, several laboratories almost simultaneously revealed that tumor suppressor p53 could directly control transcription of the miR-34 family [17–20], including miR-34a, miR-34b, and miR-34c. Since miR-34a was first linked to the p53 tumor suppressor network, its function in both biologic and pathologic processes has been intensively investigated, with demonstrations of pivotal roles of this miRNA in mediating the biological functions of p53, such as cell cycle arrest, apoptosis, and senescence [17–20]. miR-34a expression is frequently downregulated in numerous types of human malignancy, including cholangiocarcinoma, glioblastoma, leukemia, medulloblastoma, neuroblastoma, malignant peripheral nerve sheath tumor, skin, and oral squamous cell carcinoma, as well as bladder, breast, cervical, colon, colorectal, hepatocellular, non-small cell lung, ovarian, and pancreatic cancers [14, 15, 17, 21–33]. Both genetic and epigenetic mechanisms are involved in the reduction of miR-34a expression in malignant cells, for instance, inactivation of p53 and CpG methylation of the miR-34a promoter [25, 33, 34]. Enforced miR-34a expression attenuates cell proliferation and migration/invasion, inhibits tumor growth and metastasis, and induces cell cycle arrest and apoptosis [14, 15, 17, 21, 23–25, 27–31, 33], indicating that miR-34a may be a common target for therapeutic intervention of cancer. miR-34a levels also displayed prognostic significance in malignant diseases; the upregulated miR-34a expression generally correlated with better DFS/RFS/PFS/EFS of patients with solid tumors [35]. Intriguingly, miR-34a plays a dual role in chemoresistance [36–39]. miR-34a directly targets the 3’UTRs of numerous oncogenic mRNAs, including ARAF, CD44, CD117, CDK4, c-Met, cyclin D3, cyclin E2, E2F3, Fra-1, Jagged1, MCM2/5, MET, MYCN, Notch1/2, PIK3R2, PLK1, Smad4, SIRT1/6, and VEGFA, which may contribute to its tumor-suppressive role [14, 15, 18, 21, 23, 24, 26–28, 39–41]. In addition to mRNAs, our recent studies revealed that miR-34a could directly target tRNA_i_^Met^ precursors, consequently attenuating cell proliferation while inducing cell cycle arrest and apoptosis [12]. However, the role of fragments derived from miR-34a-guided tRNA_i_^Met^ cleavage in breast tumor angiogenesis remains unknown.

In this study, using two breast cancer cell lines, HCC1806, and MCF7, we discovered that PFKFB3, which is overexpressed in breast cancer cells, is a direct target of the miR-34a-guided, tRNA_i_^Met^-derived piR_019752-like fragment tRiMetF31. Enforced expression of miR-34a attenuated cancer cell migration and angiogenesis, which may be mediated, at least in part, by tRiMetF31, because ectopic tRiMetF31 inhibited migration and angiogenesis that can be blocked by tRiMetF31 siRNA. The tRiMetF31 also induced cancer cell apoptosis but had no effect on cell proliferation. We also demonstrated an inverse correlation between miR-34a expression and total tRNA_i_^Met^ levels in a large cohort of breast cancer tissues. Thus, our findings revealed that tRiMetF31 is a novel component of the miR-34a tumor suppressor network and plays a pivotal role in mediating miR-34a-induced suppression in migration and angiogenesis via targeting PFKFB3.

## Results

### miR-34a-guided, tRNA_i_^Met^-derived, piR_019752-like 31-nt fragment tRiMetF31 directly targets PFKFB3

**Fig. 1:**
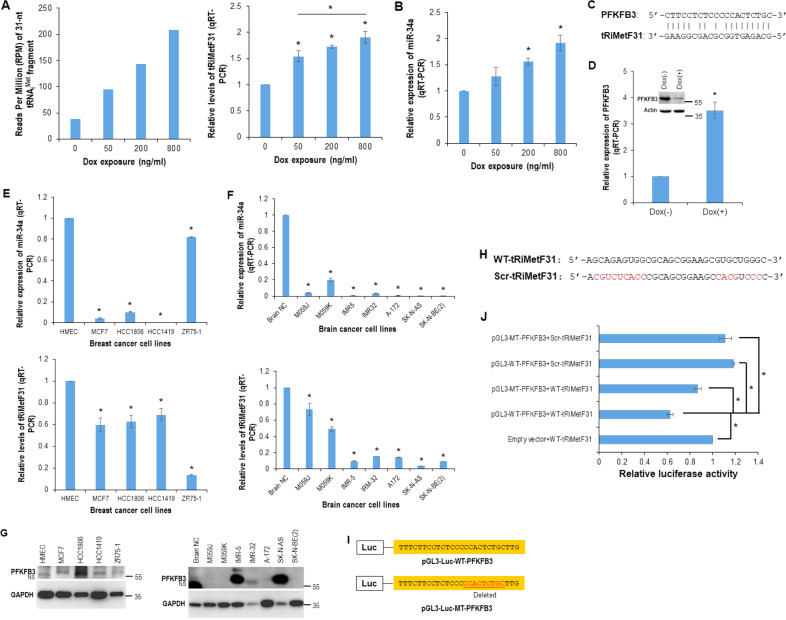
PFKFB3 is a direct target of tRNA_i_^Met^-derived fragment tRiMetF31. **A** Total RNA isolated from Dox-inducible miR-34a-expressing breast cancer HCC1806 cells was subjected to small non-coding RNA-Seq and qRT-PCR analysis of tRiMetF31, as detailed in the “Materials and methods”. **B** Total RNA isolated from Dox-inducible miR-34a-expressing breast cancer HCC1806 cells was subjected to qRT-PCR analysis of miR-34a. **C** Predicted binding motif between tRiMetF31 and *PFKFB3* 3’UTR. **D** PFKFB3 protein and mRNA levels in Dox(−) and Dox(+) HCC1806 cells were measured by Western blot analysis and qRT-PCR. **E**, **F** Total RNA isolated from the indicated breast (**E**) and brain (**F**) cancer cell lines was subjected to qRT-PCR analyses using a primer set for either miR-34a or tRiMetF31. **G** Whole cellular lysates prepared from either breast (left panel) or brain (right panel) cancer cell lines were subjected to Western blot analysis using antibody against PFKFB3. **H** Sequences of wild-type and scrambled tRiMetF31 used in the luciferase assay. **I** Sequences of wild-type and mutant PFKFB3 luciferase reporters used in the luciferase assay. **J** Co-transfection and luciferase assays were performed using Lipofectamine 3000 and a dual-luciferase reporter assay kit as detailed in the “Materials and methods”. * indicates *P* < 0.05.

We recently identified tRNA_i_^Met^ precursors as a direct target of tumor suppressor miR-34a and demonstrated that the targeted suppression of tRNA_i_^Met^ levels attenuates cell proliferation while inducing cell cycle arrest and apoptosis [12]. To screen the miR-34a-guided tRMA_i_^Met^-derived fragment(s) that may contribute to the miR-34a-mediated tumor suppression, we performed a small-RNA-Seq analysis using RNA samples isolated from the breast cancer cell line HCC1806 with doxycycline (Dox)-inducible hsa-miR-34a expression [12]. The RNA-Seq analysis identified a 31-nt tRNA_i_^Met^-derived fragment, tRiMetF31. The level of tRiMetF31 expression increased in response to doxycycline in a dose-dependent manner (Fig. 1A, left panel) and positively correlated with Dox-inducible miR-34a expression (Fig. 1B). The RNA-Seq data were validated by qRT-PCR using primer specific for tRiMetF31 (Fig. 1A, right panel). Homology analysis indicated that the tRiMetF31 sequence is completely identical to rat piR_000194 (Supplementary Fig. S1A), while having only one base difference with human piR_019752 (Supplementary Fig. S1B) [42].

We next aimed to identify the potential target(s) of tRiMetF31. Bioinformatic analysis predicted that 6-phosphofructo-2-kinase/fructose-2,6-bisphosphatase 3 (PFKFB3), a potent regulator of glycolysis [43], is a potential target of tRiMetF31 (Fig. 1C). Using Dox-inducible hsa-miR-34a-expressing HCC1806 cells as a model system, we analyzed the correlation between miR-34a and PFKFB3 expression levels. As expected, although the PFKFB3 mRNA level increased, its protein level remarkably decreased in response to Dox exposure, and this was positively correlated with Dox-inducible miR-34a expression (Fig. 1D, B). To further establish the relationship between miR-34a and tRiMetF31 and PFKFB3, we measured their levels in several breast cancer and brain cancer cell lines, with HMEC cells or normal brain tissue serving as a control. The qRT-PCR showed that miR-34a was significantly downregulated in both breast cancer and brain cancer cell lines as compared to HMEC cell line or normal brain tissue (*P* < 0.01, Fig. 1E, F, upper panels). As expected, tRiMetF31 levels were also lower in all cancer cell lines as compared to HMEC or normal brain tissue (Fig. 1E, F, lower panels), which positively correlated with miR-34a expression levels, supporting our previous finding—miR-34a-guided tRNA_i_^Met^ cleavage [12]. Interestingly, as a predicted target of tRiMetF31, PFKFB3 was upregulated in all breast cancer cell lines (Fig. 1G, left panel) and overexpressed in three out of the seven brain cancer cell lines tested (Fig. 1G, right panel). As expected, the upregulated PFKFB3 levels were negatively correlated with the tRiMetF31 levels in these cell lines (Fig. 1E, F).

To confirm that tRiMetF31 could functionally target PFKFB3, we generated a luciferase PFKFB3 reporter construct and performed luciferase assays using synthetic wild-type or scrambled tRiMetF31 (WT-tRiMetF31 or Scr-tRiMetF31; Fig. 1H, I). The luciferase assay showed that WT-tRiMetF31 reduced the luciferase activity of the reporter construct harboring wild-type PFKFB3 (WT-PFKFB3, Fig. 1J); this reduction was completely abolished by either synthetic Scr-tRiMetF31 or deletion mutant PFKFB3 (MT-PFKFB3) reporter construct or both (Fig. 1J).

**Fig. 2:**
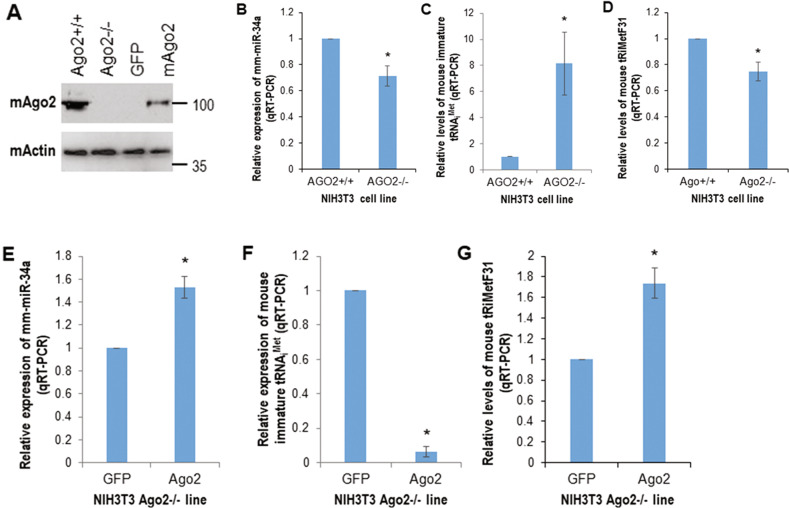
AGO2 is a key molecule in regulating the expression levels of miR-34a and tRiMetF31. **A** Whole cellular lysates prepared from mouse NIH3T3 AGO2 wild-type (Ago2^+/+^), AGO2 knockdown (Ago2^−/−^), and Ago2^−/−^ cells transfected with GFP or mouse Ago2 (mAgo2) were subjected to western blot analysis using antibody to mouse Ago2. **B**–**D** Total RNA isolated from Ago2^+/+^ and Ago2^−/−^ cells was subjected to qRT-PCR analysis using primers for mm-miR-34a, immature tRNA_i_^Met^ and tRiMetF31. **E**–**G** Total RNA isolated from GFP and mAgo2 cells was subjected to qRT-PCR analysis using primers for mm-miR-34a, immature tRNA_i_^Met^, and tRiMetF31. * indicates *P* < 0.05.

To further establish the relationship between miR-34a and tRiMetF31 in another species, we used mouse NIH3T3 AGO2 wild-type (Ago2^+/+^) and AGO2 mutant (Ago2^−/−^) cells as a model system, transiently transfected Ago2^−/−^ cells with either plasmid expressing mouse AGO2 or empty vector (GFP), and determined levels of mm-miR-34a, immature tRNA_i_^Met^ and tRiMetF31 using qRT-PCR. Western blot analysis and qRT-PCR showed that knockdown of AGO2 (Fig. 2A) caused a reduction in mm-miR-34a and tRiMetF31 levels, while it increased the level of immature tRNA_i_^Met^ expression (Fig. 2B–D). Conversely, the ectopic mouse AGO2 (mAgo2, Fig. 2A) restored the expression levels of mm-miR-34a and tRiMetF31, but attenuated the expression of immature tRNA_i_^Met^ (Fig. 2E–G). Taken together, these results suggest that overexpressed PFKFB3 in breast cancer cells is a direct target of the miR-34a-guided, tRNA_i_^Met^-derived piR_019752-like fragment tRiMetF31.

### TRiMetF31 slightly induces apoptosis but does not influence the proliferation of breast cancer cells

**Fig. 3:**
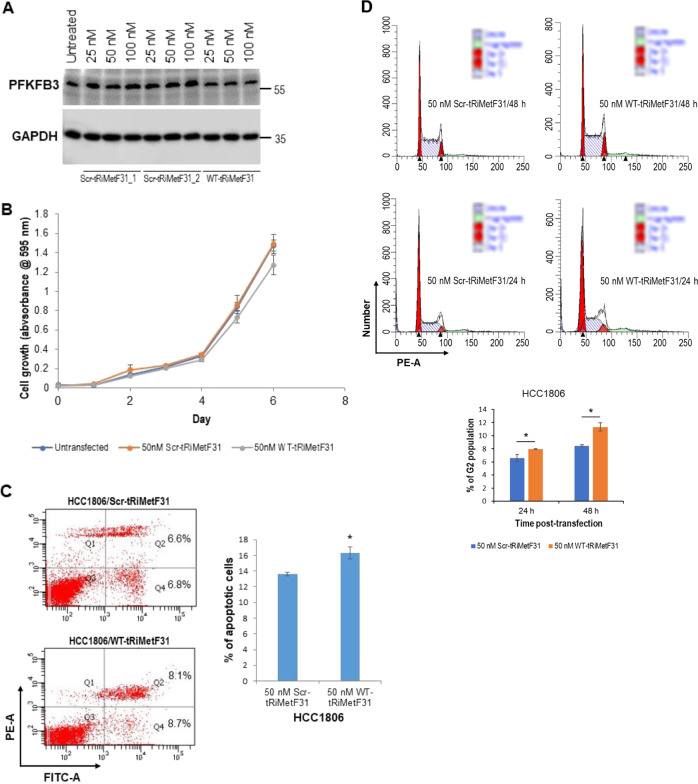
Ectopic tRiMetF31 induced apoptosis and cell cycle arrest, while having no effect on the proliferation of HCC1806 cells. **A** HCC1806 cells were transfected with either Scr-tRiMetF31_1 or Scr-tRiMetF31_2 or WT-tRiMetF31; at 48 h after transfection, whole cellular lysates were prepared and subjected to western blot analysis using antibody against PFKFB3. **B** At 24 h after transfection, cells were replated in 96-well plates, and MTT assay was performed as detailed in the “Materials and methods”. **C**, **D** HCC1806 cells were transfected with either 50 nM WT-tRiMetF31 or 50 nM Scr-tRiMetF31_2; at 24 h and/or 48 h after transfection, cells were harvested for apoptosis and cell cycle analyses, as detailed in the “Materials and methods”. * indicates *P* < 0.05.

**Fig. 4:**
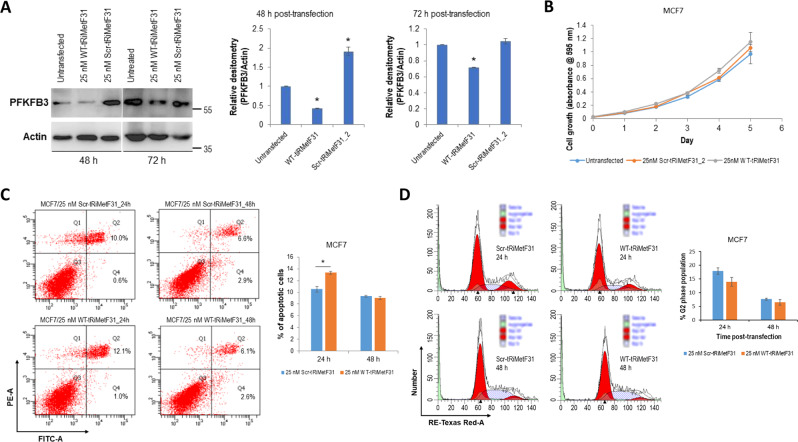
Ectopic tRiMetF31 induced apoptosis while having no effect on the proliferation of MCF7 cells. **A** MCF7 cells were transfected with either 25 nM Scr-tRiMetF31_2 or 25 nM WT-tRiMetF31; at 24 h and 48 h after transfection, whole cellular lysates were prepared and subjected to Western blot analysis using antibody against PFKFB3, as detailed in the “Materials and methods”. **B** At 24 h after transfection, cells were replated in 96-well plates, and MTT assay was performed as detailed in the “Materials and methods”. **C**, **D** MCF7 cells were transfected with either 25 nM WT-tRiMetF31 or 25 nM Scr-tRiMetF31_2; at 24 h and/or 48 h after transfection, cells were harvested for apoptosis and cell cycle analyses, as detailed in the “Materials and methods”. * indicates *P* < 0.05.

Using two breast cancer cell lines, HCC1806 and MCF7, as a model system, we next explored the effect of tRiMetF31 on breast cancer cell biology. HCC1806 cells were transiently transfected with the indicated concentration of either Scr-tRiMetF31_1 or Scr-tRiMetF31_2 or WT-tRiMetF31. Western blot analysis indicated that at 48 h after transfection, PFKFB3 expression was weakened by three different concentrations of WT-tRiMetF31 tested (Fig. 3A), whereas its expression was not affected by both Scr-tRiMetF31_1 and Scr-tRiMetF31_2. Although 50 nM of tRiMetF31 induced apoptosis and G2 cell cycle arrest (Fig. 3C, D), it had no effect on cell proliferation (Fig. 3B). Similarly, 25 nM WT-tRiMetF31 caused downregulation of PFKFB3 and induced apoptosis in MCF7 cells (Fig. 4A, C); however, it had no effect on cell proliferation and cell cycle (Fig. 4B, D). Also, we noted the transient induction in PFKFB3 expression caused by Scr-tRiMetF31, which could be in part due to the effects of Lipofectamine 3000 reagent. Indeed, previous evidence has demonstrated that transfection reagents could induce cellular stress [44], while suppression of PFKFB3 may enhance endoplasmic reticulum (ER) stress [45], supporting the role of PFKFB3 in cellular stress. These results suggest that tRiMetF31 could slightly induce apoptosis of breast cancer cells, while having no effect on cell proliferation.

### tRiMetF31 inhibits migration and angiogenesis

**Fig. 5:**
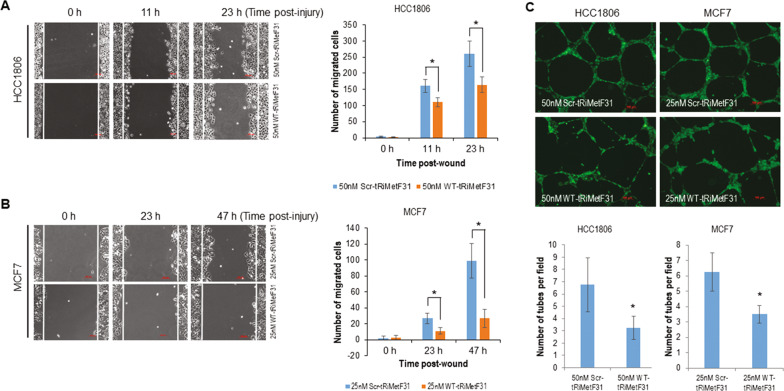
Ectopic tRiMetF31 inhibited the migration and angiogenesis of breast cancer cells. **A**, **B** HCC1806 (A) and MCF7 (B) cells grown in 6-well plates were transfected with either 50 nM or 25 nM WT-tRiMetF31 or Scr-tRiMetF31_2; at 22 h after transfection, the cells were treated with mitomycin C for 2 h, and a wound-healing assay was then performed. **C** Tube-formation assay was carried out using 50% of 48 h conditioned medium from either HCC1806 or MCF7 cells transfected with either 50 nM or 25 nM WT-tRiMetF31 or Scr-tRiMetF31_2 as detailed in the “Materials and methods”. * indicates *P* < 0.05.

Recently, strong and growing evidence has highlighted the crucial role of PFKFB3 in tumor angiogenesis and metastasis [46–48]. Thus, we examined the contributing role of tRiMetF31-triggered downregulation of PFKFB3 in breast cancer migration and angiogenesis using HCC1806 and MCF7 cell lines. The wound-healing assay showed that 50 nM WT-tRiMetF31 suppressed HCC1806 cell migration (Fig. 5A). Similar effect was also found in MCF7 cells in response to 25 nM WT-tRiMetF31 (Fig. 5B). As expected, tube-formation assay indicated that angiogenesis was profoundly inhibited by 50% (v/v) conditioned medium from either HCC1806 transfected with 50 nM WT-tRiMetF31 or MCF7 transfected with 25 nM WT-tRiMetF31 (Fig. 5C).

**Fig. 6:**
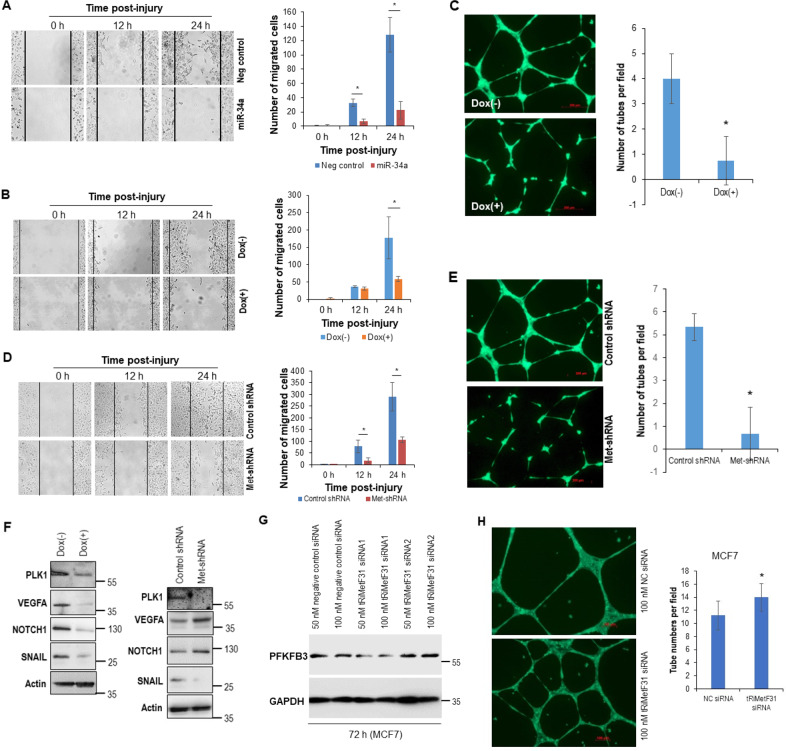
Both ectopic miR-34a and tRNA_i_^Met^ knockdown suppressed the migration and angiogenesis of breast cancer cells. **A** HCC1806 cells grown in six-well plates were transfected with either 33 nM miR-34a or 33 nM AllStars negative control; at 22 h after transfection, the cells were exposed to mitomycin C for 2 h, and a wound-healing assay was then performed. **B** Dox-inducible miR-34a-expressing HCC1806 cells grown in six-well plates were incubated in either Dox-containing or Dox-free medium for 72 h; at 2 h before wound, the cells were treated with mitomycin C, a wound-healing assay was then performed. **C** Tube-formation assay was performed using 50% conditioned medium from Dox(-) or Dox(+) cells, as detailed in the “Materials and methods”. **D** HCC1806 cells stable expressing either tRNA_i_^Met^ shRNA (Met-shRNA) or control shRNA were seeded in six-well plates and exposed to mitomycin C for 2 h before generating a wound. A wound-healing assay was then performed. **E** Tube-formation assay was carried out using conditioned medium from either Met-shRNA- or control shRNA-expressing cells, as detailed in the “Materials and methods”. **F** Whole cellular lysates prepared from either Dox(-) or Dox(+) cells or Met-shRNA- or control shRNA-expressing cells were subjected to Western blot analysis using antibodies to PLK1, VEGF, NOTCH1, and SNAIL, actin served as a loading control. **G** MCF7 cells were transiently transfected with either 50 nM or 100 nM tRiMetF31 siRNA1 or tRiMetF31 siRNA2 or AllStars negative control siRNA; 72 h after transfection, whole cellular lysates were prepared and subjected to Western blot analysis using antibody to PFKFB3. **H** Tube-formation assay was carried out using 50% conditioned medium from either tRiMetF31 siRNA2- or negative control siRNA-transfected cells, as detailed in the “Materials and methods”. * indicates *P* < 0.05.

To confirm the contributing role of tRiMetF31 in miR-34a-mediated biological and pathological processes, we assessed the role of miR-34a in breast cancer cell migration and angiogenesis. The wound-healing assay showed that transiently transfected and inducibly expressed miR-34a suppressed HCC1806 cell migration (Fig. 6A, B). Angiogenesis was attenuated by 50% (v/v) conditioned medium from Dox-inducible miR-34a-expressing HCC1806 cells (Fig. 6C). These results are consistent with previous studies [16, 49], supporting the contributing role of tRiMetF31 in miR-34a-mediated suppression of both migration and angiogenesis. Production of tRiMetF31 from cleavage of tRNA_i_^Met^ precursor may lead to a reduction in tRNA_i_^Met^. To determine whether the reduction of tRNA_i_^Met^ also affects migration and angiogenesis, we examined the impact of tRNA_i_^Met^-knockdown on these processes using a previously established HCC1806 cell line stably expressing tRNA_i_^Met^-shRNA (Met-shRNA, ref. [12]). As expected, knockdown of tRNA_i_^Met^ inhibited HCC1806 cell migration (Fig. 6D), and angiogenesis was also attenuated by 50% (v/v) conditioned medium from HCC1806 cells stably expressing Met-shRNA (Fig. 6E).

To explore other molecules that may also play a role in the angiogenic and invasive suppression, mediated by the ectopic expression of miR-34a and the knockdown of tRNA_i_^Met^, 2D electrophoresis (2DE)/MALDI-TOF analysis was performed. The 2DE/MALDI-TOF analysis indicated downregulation of Polo-like kinase 1 (PLK1) in both cell lines (Supplementary Fig. S2), which was validated by western blot analysis (Fig. 6F). Because vascular endothelial growth factor A (VEGFA) and Drosophila Notch homolog 1 (NOTCH1) are two well-defined angiogenic molecules that have been demonstrated to be direct targets of miR-34a [14, 41], we also measured the expression of these two molecules in both enforced miR-34a expression and tRNA_i_^Met^ knockdown HCC1806 cells. Western blot analysis showed that the expression of VEGFA and NOTCH1 was downregulated in HCC1806 cells inducibly expressing miR-34a (Fig. 6F, left panel), supporting previous discoveries [14, 41]. However, VEGFA and NOTCH1 were upregulated in tRNA_i_^Met^ knockdown HCC1806 cells (Fig. 6F, right panel). Interestingly, the epithelial-to-mesenchymal transition (EMT)-associated transcription factor SNAIL was downregulated in both enforced miR-34a expression and tRNA_i_^Met^ knockdown HCC1806 cells (Fig. 6F).

To further validate the suppressive role of tRiMetF31 in PFKFB3-mediated angiogenesis, we attempted to functionally inhibited tRiMetF31 in MCF7 cells using siRNA. To date, no validated siRNAs targeting tRiMetF31 are commercially available. Two siRNAs used in this study were designed using Dharmacon siRNA design to target PFKFB3. Western blot analysis showed that 100 nM tRiMetF31 siRNA2 elevated PFKFB3 expression compared to 100 nM negative control siRNA (Fig. 6G). The observed downregulation of PFKFB3 induced by siRNA1 may be an off-target effect. The mechanism involved may be complex. siRNA1 may target the key transcription factors and/or other factors governing PFKFB3 transcription, translation, and degradation. Nonetheless, this study constitutes the first-ever attempt to functionally inhibit tRiMetF31 in MCF7 cells using siRNA. Most importantly, the tube-formation assay indicated that angiogenesis was mildly enhanced by tRiMetF31 knockdown (Fig. 6H). Taken together, these results suggest that tRiMetF31 suppresses the migration and angiogenesis of breast cancer cells.

### An inverse correlation between miR-34a levels and total tRNA_i_^Met^ or PFKFB3 levels in breast cancer

**Fig. 7:**
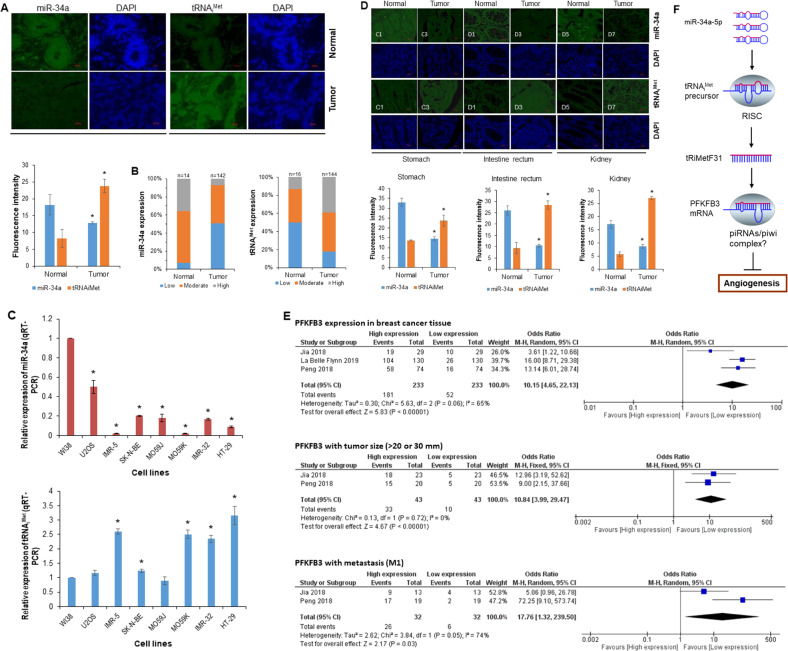
Expression of miR-34a and tRNA_i_^Met^ in cancer tissues and cell lines. **A** Presentative images for expression of miR-34a and tRNA_i_^Met^ that were determined in breast cancer tissues using FISH, as detailed in the “Materials and methods”. **B** Statistic analysis of miR-34a and tRMA_i_^Met^ FISH staining. **C** Total RNA isolated from breast and brain cancer cell lines was subjected to qRT-PCR analysis using primers for either miR-34a or total tRNA_i_^Met^. **D** Representative images for expression of miR-34a and tRNA_i_^Met^ that was determined in stomach, kidney, and intestinal cancer tissues using FISH as detailed in the “Materials and methods”. **E** A meta-analysis of PFKFB3 expression in breast tumor tissues. **F** Diagram of the potential role of miR-34a/tRiMetF31/PFKFB3 axis in angiogenic suppression. * indicates *P* < 0.05.

Our previous studies indicated that miR-34a expression levels were inversely correlated with tRNA_i_^Met^ levels in breast cancer cell lines [12]. To further establish this relationship, we analyzed miR-34a and total tRNA_i_^Met^ (both precursor and mature molecules) expression levels in a large-scale breast cancer tissue array using FISH, and subsequently performed a correlation analysis between the expression of these two molecules. The FISH analysis showed that miR-34a was downregulated in tumor tissues (51%, *n* = 142), whereas tRNA_i_^Met^ was upregulated in tumor tissues (39%, *n* = 144) compared with normal tissues (Fig. 7A, B; Spearman correlation = 0.99, *P* < 0.05). To further confirm this inverse correlation, we determined the expression levels of miR-34a and tRNA_i_^Met^ in other carcinoma cell lines and tissue sections. Real-time RT-PCR and FISH analyses indicated the same correlation between downregulated miR-34a and elevated tRNA_i_^Met^ in malignant glioma cell lines (M059J and M059K), neuroblastoma cell lines (IMR-5 and IMR-32), a colon adenocarcinoma cell line (HT-29), and tissue sections of stomach, rectal, and kidney carcinomas (Fig. 7C, D). Regarding PFKFB3 expression in breast cancer tissues, numerous studies have been done with immunohistochemical staining [50–52], and we therefore performed a meta-analysis using data from these studies. The meta-analysis indicated that PFKFB3 was overexpressed in breast cancer tissues, which was significantly correlated with tumor size and metastasis (Fig. 7E). TCGA data analysis showed that two out of nine tRNA-Met fragments were expressed in breast cancer, which were 5P_tRNA-iMet-CAT-1-3 and ts-63 (Supplementary Table S1). Interestingly, ts-63 was significantly downregulated in breast cancers (Supplementary Fig. S3); however, tRiMetF31 was undetectable. Collectively, these results suggest that the downregulation of miR-34a and the upregulation of tRNA_i_^Met^ may be common events in the development of human cancers.

## Discussion

As the most abundant RNA in human cells, tRNA primarily functions as the adaptor of amino acids, reading genetic codes and translating genetic information from mRNAs to the sequence of amino acids. In normal cells, the abundance of tRNAs is tightly controlled both transcriptionally and post-transcriptionally by tumor suppressors, ribonucleases, and tRNA modification enzymes. The tumor suppressors RB, p53, ARF, PTEN, and BRCA1 can modulate tRNA transcription by inhibiting RNA polymerase I and/or III [53–55]. Inactivation of these tumor suppressors caused by genetic and epigenetic factors leads to deregulation of RNA polymerases I and III, eventually resulting in tRNA overexpression and progression of human malignancies [56–58].

In the last decade, a new class of tRNA-derived small non-coding RNAs has been identified and linked to cancer. In this study, we uncovered a novel 31-nt miR-34a-guided, tRNA_i_^Met^-derived, piR_019752-like fragment tRiMetF31 and revealed a crucial role of this fragment in suppressing the migration and angiogenesis of breast cancer via silencing PFKFB3. We show that the levels of both miR-34a and tRiMetF31 were increased in miR-34a-inducible expression breast cancer cell line HCC1806 in response to doxycycline in a dose-dependent meaner. miR-34a and tRiMetF31 were downregulated in all breast and brain cancer cell lines examined, and there was a positive relationship in the expression between miR-34a and tRiMetF31.

We also show that PFKFB3 is a direct target of tRiMetF31. PFKFB3 was overexpressed in all breast cancer cell lines and in 42.9% of brain cancer cell lines (*n* = 7) tested, which may be attributed to the downregulation of tRiMet31 in these cell lines.

Interestingly, three out of four neuroblastoma cell lines (IMR-5, IMR-32, and SK-N-AS) overexpressed PFKFB3, which may be attributed to the downregulation of tRiMetF31 (Fig. 1F). Although tRiMetF31 was also downregulated in three glioblastoma cell lines (A-172, M059J, and M059K), PFKFB3 was undetectable in all three glioblastoma lines. This may suggest that tRiMetF31-mediated silencing of PFKFB3 is cell type- or tissue-dependent and needs to be further explored in this context.

Furthermore, we noted that although miR-34a is downregulated in all breast and brain cancer cell lines examined, the tRiMetF31 expression in some cases is not downregulated to the same extent as miR-34a, which may be due to the difference in cell content of different cancer cell lines. While this observation, in our opinion, does not affect the conclusion that tRiMetF31 levels are positively correlated with the miR-34a expression levels in cancer cell lines tested, more studies are needed in the future to analyze this relationship in the context of other cell lines and tumor types.

tRNA cleavage products are well-known biomarkers of cancer. However, the tRNA-derived fragments (tRFs) have not drawn much attention until the identification of a new class of small non-coding RNAs derived from tRNAs by high-throughput profiling. Accumulating evidence has shown a dysregulation of tRNA-derived fragments in human malignancies, which may function as either an oncogene or tumor suppressor to play a contributing role to tumorigenesis [59–61]. Numerous tRFs have been shown to be abundantly expressed in estrogen receptor-positive breast cancer and androgen receptor-positive prostate cancer and to enhance cancer cell proliferation [59]. Knockdown of a specific tRF induced apoptosis of hepatocellular carcinoma, both in vitro and in vivo [60]. Several tRFs also suppress breast cancer progression by displacing oncogenic transcripts from the RNA-binding protein YBX1 [60].

Here we show that tRiMetF31 could slightly induce apoptosis of breast cancer cells, while having no effect on cell proliferation. This is an interesting finding, as cells can share common molecules to regulate both cell proliferation and cell death, such as tumor suppressors p53 and RB, cyclin-dependent kinases and their regulators. These molecules not only function in cell cycle/proliferative pathways, but also contribute to apoptosis. Interestingly, in the last decade, growing evidence has indicated that the apoptotic cells could promote the proliferation of surrounding cells (apoptosis-induced proliferation) due to the mitogenic signals released from the apoptotic cells. Hence, there may be a balance between the proliferative inhibition triggered by cell cycle arrest and apoptosis-induced proliferation, eventually contributing to cell growth rate. Further studies are needed in the future to dissect the roles of tRiMetF31 in detail.

In this study, we provide evidence of a pivotal role for Ago2 in regulating the expression of miR-34a and immature tRNA_i_^Met^ and the production of tRiMetF31 using loss-of-function and gain-of-function approaches. Importantly, tRiMetF31 markedly inhibited migration and angiogenesis, as well as induced apoptosis of two breast cancer cell lines, HCC1806, and MCF7, by silencing PFKFB3, although it had no effect on cancer cell proliferation. The anti-angiogenic effect of tRiMetF31 was reversed by tRiMetF31 knockdown. Mechanically, tRiMetF31 may target PFKFB3 through Argonaute- and/or Piwi-mediated silencing, since both have been shown to interact with tRFs [62–64]. PFKFB3 is an allosteric activator of 6-phosphofructo-1-kinse (PFK-1), which converts fructose-6-phosphate to fructose-1,6-biphosphate, a rate-limiting reaction of the glycolytic flux. PFKFB3 may function as an oncogene in tumorigenesis, since the overexpression of PFKFB3 has been demonstrated in numerous malignancies, including breast cancer [65]. PFKFB3 knockdown inhibited cancer cell proliferation in vitro and attenuated tumor growth/metastasis in animal models [65–67]. Recently, several lines of evidence have also indicated a key role for PFKFB3 in driving angiogenesis [46], which supports our findings.

Although our findings revealed tRiMetF31 to be a crucial component of the miR-34a tumor suppressor network, we noted that the enforced expression of miR-34a itself also weakened the expression of PLK1, SNAIL, VEGFA, and NOTCH1, which are validated targets of miR-34a, that may also contribute to miR-34a-induced suppression in migration and angiogenesis. We revealed that tRNA_i_^Met^ may also have a contributing role in angiogenesis, since knockdown of tRNA_i_^Met^ attenuated angiogenesis, although the expression of VEGFA and NOTCH1 was even enhanced. The mechanism involved is currently unclear and needs to be investigated in the future.

Due to restrictions on both technology and the availability of breast cancer tissue samples, we are currently unable to determine tRiMetF31 levels in a large cohort of tissue samples using either qRT-PCR or FISH. tRiMetF31 was also undetectable by analysis of the TCGA database, which is probably due to its low copy number [68, 69]. Instead, we showed that miR-34a was downregulated, while total tRNA_i_^Met^ (both precursor and mature) was upregulated in breast cancer tissues, with an inverse correlation between them. This was supported by findings from non-breast cancer cell lines and tissues. Interestingly, the meta-analysis showed an overexpression of PFKFB3 in breast cancer tissues, which was significantly correlated with tumor size and metastasis. We also noted an inverse correlation between miR-34a levels and PFKFB3 expression in breast cancer tissues, while no evidence supports PFKFB3 to be a direct target of miR-34a, which may reflect the existence of a bridge molecule (such as tRiMetF31) that functionally links the changes of these molecules.

While knockdown of tRNA_i_^Met^ led to downregulation of many proteins induced by tRNA_i_^Met^ shRNA, quite unexpectedly, some proteins, including VEGFA, were upregulated in breast cancer HCC1806 cells in response to tRNA_i_^Met^ knockdown. The mechanism involved is still unclear and needs to be further elucidated in the future. Perhaps there is a mechanism to guarantee the synthesis of key proteins for cancer cell survival in priority under global tRNA_i_^Met^ knockdown stress.

In conclusion, the novel miR-34a-guided, tRNA_i_^Met^-derived fragment tRiMetF31, which was downregulated in cancer cell lines, suppresses migration and angiogenesis of breast cancer cells via targeting PFKFB3 (Fig. 7F), and that can be reversed by tRiMetF31 knockdown, highlighting a crucial anti-angiogenic role of miR-34a/tRiMetF31/PFKFB3 axis in breast cancer progression. tRiMetF31 may represent a target molecule for therapeutic intervention.

## Materials and methods

### Cell culture

Human mammary epithelial cells (HMEC) purchased from Invitrogen were cultured in HuMEC Basal Serum-Free Medium containing HuMEC Supplement (Invitrogen); breast cancer cell lines ZR75-1, HCC1419, and HCC1806 obtained from ATCC were grown in ATCC-formulated RPMI-1640 Medium (ATCC) supplemented with 10% fetal bovine serum (FBS) and 1% penicillin/streptomycin (P/S); MCF7 cells purchased from ATCC were cultured in Dulbecco’s Modified Eagle’s Medium (DMEM)/F12 (HyClone) supplemented with 10% FBS and 1% P/S; HEK293 cells were grown in DMEM/High Glucose (Thermo Scientific Limited) supplemented with 10% FBS and 1% P/S. Human glioblastoma cell lines M059J, M059K, and A-172 and neuroblastoma cell line SK-N-BE(2) were purchased from ATCC, while IMR-5, IMR-32, and SK-N-AS neuroblastoma cell lines were gift lines kindly provided by Dr. Aru Narendran (Arnie Charbonneau Cancer Institute, University of Calgary, Calgary, Canada). M059J and M059K cells were grown in a 1:1 mixture of DMEM and Ham’s F12 medium with 2.5 mM L-glutamine adjusted to contain 15 mM HEPES, 0.5 mM sodium pyruvate, and 1.2 g/L sodium bicarbonate supplemented with 0.05 mM non-essential amino acids (NEAA), 10% FBS, and 1% P/S. A-172 cells were grown in an ATCC-formulated DMEM supplemented with 10% FBS and 1% P/S. SK-N-BE(2) cells were grown in a 1:1 mixture of ATCC-formulated Eagle’s Minimum Essential Medium (EMEM) and F12 Medium supplemented with 10% FBS and 1% P/S. IMR-5 cells were cultured in RPMI 1604 medium supplemented with 2 mM L-Glutamine, 1% NEAA, 10% FBS and 1% S/P. IMR-32 and SK-N-AS cells were grown in EMEM medium supplemented with 10% FBS and 1% P/S. SK-N-AS cells were cultured in DMEM medium supplemented with 1% NEAA, 10% FBS, and 1% P/S. Colorectal adenocarcinoma HT-29 cells and osteosarcoma U2OS cells purchased from ATCC were grown in McCoy’s 5a medium supplemented with 10% FBS and 1% P/S. Human lung fibroblast WI-38 cells purchased from ATCC were cultured in EMEM supplemented with 10% FBS and 1% P/S. All cells were cultured at 37 °C in a humidified atmosphere of 5% CO_2_. Cell lines used in functional studies were treated with the mycoplasma removal reagent BM-Cyclin (Roche) to ensure mycoplasma negativity before treatment.

### RNA-Seq analysis

The doxycycline (Dox)-inducible hsa-miR-34a-expressing HCC1806 cells were generated previously [12]. The Dox-inducible hsa-miR-34a-expressing HCC1806 cells were grown in RPMI-1640 medium and treated with the indicated concentration of doxycycline for 72 h. Total RNA isolated using TRIzol reagent (Invitrogen) was then treated with RNase-free DNase I (ThermoFisher Scientific), followed by RNA re-isolation with TRIzol reagent, according to the manufacturers’ instructions. The RNA samples were then subjected to RNA-Seq analysis. The sequencing libraries were prepared using TruSeq Small RNA Library Prep Kit (Illumina, San Diego, CA) according to the manufacturer’s instructions. The sequencing was conducted by GenomeQuebec (https://www.genomequebec.com/) using the Illumina HiSeq2000 system. The libraries were sequenced for 50 cycles with a single-end configuration. The initial assessment of sequencing quality done with FastQC (https://www.bioinformatics.babraham.ac.uk/projects/fastqc/) confirmed high base qualities. FastQC analysis confirmed the presence of Illumina adapters in the read sequences due to read-through resulting from the short length of small-RNA-derived fragments. The reads were trimmed of adapters with cutadapt v.1.10.

To estimate the expression of MetCAT tRNA fragment, corresponding sequences were downloaded using UCSC table browser (hg19 genome assembly) [70]. The sample reads mapped to MetCAT tRNA sequences using bowtie v.1.1.2 [71] with no mismatches allowed. Mapped sequences were counted and scaled to the library size as a percent from the total; mapping with 1 or 2 mismatches produced similar results (data not shown). The levels of full-length tRNA-Met transcripts were determined by bowtie mapping sequencing reads that did not contain adapters (50–51 bp) and counting valid alignments.

The length distribution profiles were obtained by extracting relevant results for FastQC generated data and scaling to the library size as reads per million (RPMs). The library composition results were generated as follows: (1) trimmed reads were mapped to hg19 genome, reads with no match were counted and removed from the further analysis; (2) reads with valid matches to the genome were mapped in a sequential fashion to various genomic categories, reads mapping to a given dataset were counted and removed, while the remaining reads were mapped to the next dataset. The sequential mapping adhered to the following precedence: miRNA ← piRNA ← snoRNA ← rRNA ← tRNA ← CDS ← Promoters ← 5’UTR ← 3’UTR ← Introns ← repeats. The datasets for various genomic categories were downloaded from UCSC browser [72], Ensembl Biomart [73], miRBase [74] and GenBank [75]. All of the mappings in library composition profile analysis were done using bowtie allowing for 1 mismatch with –best option enables.

### Quantitative real-time RT-PCR (qRT-PCR)

To measure hsa-miR-34a levels, total RNA was isolated from the indicated cells using TRIzol reagent (Invitrogen) and subjected to qRT-PCR using an miScript II RT Kit (QIAGEN) and QuantiTect SYBR Green PCR Master Mix (GIAGEN) with a primer set for hsa-miR-34a and mm-miR-34a per the manufacturers’ instructions. Human/mouse *RNU6-2* served as a loading control. To determine the total tRNA_i_^Met^ levels, total RNA was isolated using TRIzol reagent, and qRT-PCR was performed as described previously [12]. The experiments were done in triplicate. To measure *PFKFB3* mRNA levels, total RNA was isolated from the indicated cells using TRIzol reagent and subjected to qRT-PCR using an iScript Select cDNA Synthesis kit (Bio-Rad) and SsoFast EvaGreen Supermix (Bio-Rad) with a primer set for PFKFB3 according to the manufacturers’ instructions. The *glyceraldehyde-3-phosphate dehydrogenase* (*GAPDH*) gene was used as an internal control. To determine tRiMetF31 levels, total RNA was isolated using TRIzol reagent, and qRT-PCR was performed as described previously [76]. Briefly, 6 μg of total RNA samples were separated by electrophoresis on a 15% denaturing polyacrylamide gel (1X DEPC-treated TBE buffer, 15% acrylamine/bis-acrylamide [19:1], 6.5 M urea, 0.08% ammonium persulfate, 0.04% N,N,N’,N’-tetramethyl-ethylenediamine), 80 V for 4 h; after electrophoresis, gel was stained with Safe-Red (Applied Biological Materials Inc.) and the RNA was visualized under UV; a piece of gel containing 15–150 nt RNA was cut off and the RNA was purified using ZR small-RNA PAGE Recovery Kit (ZYMO Research) according to the manufacturer’s instruction. The recovered small RNA was then polyadenylated using Poly(A) polymerase (Ambion) and purified with TRIzol reagent (Invitrogen) according to the manufacturers’ instructions. Reverse transcription for 31 nt tRiMetF31 qPCR was performed using iScript^TM^ Select cDNA Synthesis kit (Bio-Rad) with RTQ primer [76], and the qRT-PCR was carried out using SsoFast^MT^ EvaGreen Supermix (Bio-Rad) with tiMetF-SP and RTQ-UNIr primers according to the manufacturer’s instructions. The reverse transcription for the RNU6–2 loading control was performed using the miScript II RT Kit (QIAGEN), and the qRT-PCR was then performed using miScript SYBR^®^ Green PCR Kit (QIAGEN) with the RNU6–2 primer set according to the manufacturer’s instructions. Primers used in this study include RTQ primer: 5’-CGA ATT CTA GAG CTC GAG GCA GGC GAC ATG GCT GGC TAG TTA AGC TTG GTA CCG AGC TCG GAT CCA CTA GTC CTT TTT TTT TTT TTT TTT TTT TTT TTG C-3’; tRiMetF31 specific primer (tRiMetF-SP): 5’-AAG CGT GCT GGG CAA AAA-3’; RTQ-UNIr: 5’-CGA ATT CTA GAG CTC GAG GCA GG-3’.

### Western blot analysis

The indicated cells were washed twice with ice-cold PBS and lysed in a radioimmunoprecipitation assay buffer (RIPA). In total, 30–100 μg of protein per sample was separated on a 10% SDS-PAGE and electrophoretically transferred to a polyvinylidene difluoride (PVDF) membrane (Amersham Hybond^®^ P, GE Healthcare) at 4 °C for 1.5 h. Blots were incubated for 1 h with 5% nonfat dry milk to block nonspecific binding sites, and then they were incubated overnight at 4 °C with polyclonal/monoclonal antibodies specific to AGO2 (Cat# ab32381), NOTCH1 (Cat# ab52627), VEGFA (Cat# ab46154) (all from Abcam) or PLK1 (Cat# 4513), PFKFB3 (Cat# 13123), SNAIL (Cat# 3895) (all from Cell Signaling Technology). The immunoreactivity was detected using a peroxidase-conjugated antibody and was visualized by an ECL Plus Western Blotting Detection System (GE Healthcare). The blots were stripped before reprobing with an antibody against actin (Abcam) or GAPDH (Santa Cruz Biotechnology).

### Construction of a PFKFB3 luciferase reporter

A PFKFB3 luciferase reporter bearing either a predicted wild-type or mutant tRiMetF31-binding site was generated by synthesizing the following oligos: PFKFB3-WT1: 5′-[Phos] CTA GAC TTT TTT TTT TTC CTT TTC CAA CCT GTT TCT TCC TCT CCC CCA CTC TGC TTG AAA GAC G-3’, PFKFB3-WT2: 5’-[Phos] AAT TCG TCT TTC AAG CAG AGT GGG GGA GAG GAA GAA ACA GGT TGG AAA AGG AAA AAA AAA AAG T-3’; PFKFB3-MT1: 5’-[Phos] CTA GAC TTT TTT TTT TTC CTT TTC CAA CCT GTT TCT TCC TCT CCC TTG AAA GAC G-3’ and PFKFB3-MT2: 5’-[Phos] AAT TCG TCT TTC AAG GGA GAG GAA GAA ACA GGT TGG AAA AGG AAA AAA AAA AAG T-3’. After annealing, the double-stranded oligos were cloned downstream of the luciferase gene in the pGL3-Basic vector between *Xba*I and *EcoR*I (a linker introduced by Mr. James Meservy) to generate Luc-WT-PFKFB3 and Luc-MT-PFKFB3 reporters. The sequence identity was confirmed with automated DNA sequencing.

### Luciferase assay

HEK293 cells grown to 90% confluency in six-well plates were transiently cotransfected with 100 ng of either WT-PFKFB3 or MT-PFKFB3 luciferase reporter and 5 ng of pRL-TK plasmid in combination with either 100 nM WT-tRiMetF31 or 100 nM MT-tRiMetF31 using Lipofectamine 3000 (Invitrogen), according to the manufacturer’s instruction. At 24 h after transfection, the cells were lysed in passive lysis buffer, and the relative luciferase activity was determined by the Dual-Luciferase Reporter Assay System (Promega) using a luminometer (FLUOstar Omega, BMG LABTECH) with Firefly luciferase data normalized to Renilla. The assay was performed in duplicate.

### MTT assay

Breast cancer cells HCC1806 and MCF7 grown to 90% confluency were transfected with either 50 nM WT-tRiMetF31 or 50 nM Scr-tRiMetF31_2 or 25 nM WT-tRiMetF31 or 25 nM Scr-tRiMetF31_2 using Lipofectamine 3000 (Invitrogen), according to per the manufacturer’s instructions. At 24 h after transfection, 3.0 × 10^3^ cells were plated in 96-well plates. The 3-(4,5-dimethylthiazol-2-yl)-2,5-diphenyl tetrazolium bromide (MTT) assays were performed using the Cell Proliferation Kit I (Roche Diagnostics GmbH) according to the manufacturer’s instructions. The spectrophotometric absorbance of the samples was measured at 595 nm using a microtiter plate reader (FLUOstar Omega). The synthetic RNA oligos used here include WT-tRiMetF31: 5’-AGC AGA GUG GCG CAG CGG AAG CGU GCU GGG C-3’; Scr-tRiMetF31_2 (Scr-tRiMetF31): 5’-AUA UUA CUG AUA ACG UCA AUU CUA ACA AUA A-3’.

### Apoptosis analysis

Breast cancer cells HCC1806 and MCF7 grown to 90% confluency were transfected with either 50 nM WT-tRiMetF31 or 50 nM Scr-tRiMetF31 or 25 nM WT-tRiMetF31 or 25 nM Scr-tRiMetF31 using Lipofectamine 3000 (Invitrogen), according to the manufacturer’s instructions. At 48 h after transfection, the cells were harvested for apoptosis analysis, which was performed using a BD FACSCanto™ II Flow Cytometer (BD Biosciences) with a BD Pharmingen™ V-FITC Annexin Apoptosis Detection Kit II (BD Biosciences), according to the manufacturer’s instructions.

### Cell cycle analysis

Breast cancer cells HCC1806 and MCF7 grown to 90% confluency were transfected with either 50 nM WT-tRiMetF31 or 50 nM Scr-tRiMetF31 or 25 nM WT-tRiMetF31 or 25 nM Scr-tRiMetF31 using Lipofectamine 3000 (Invitrogen), according to the manufacturer’s instructions. At 24 and 48 h after transfection, the cells were harvested for cell cycle analysis, which was carried out using a BD FACSCanto™ II Flow Cytometer (BD Biosciences) with a propidium iodide staining solution (BD Biosciences), according to the manufacturer’s instructions.

### Wound-healing assay

HCC1806 cells inducibly expressing either miR-34a or stably expressing Met-shRNA were replated in 6-well plates and incubated at 37 °C in a humidified atmosphere of 5% CO_2_ for an additional 24 h. Breast cancer cells HCC1806 and MCF7 grown to 90% confluency in 6-well plates were transfected with either 50 nM WT-tRiMetF31 or 50 nM Scr-tRiMetF31 or 25 nM WT-tRiMetF31 or 25 nM Scr-tRiMetF31 using Lipofectamine 3000 (Invitrogen) according to the manufacturer’s instructions. At 24 h after incubation/transfection, the cells were exposed to 5 μg/ml mitomycin C for 2 h prior to injury [77]. The wound-healing assay was performed as described previously [77].

### Tube-formation assay

Human umbilical vein endothelial cells (HUVECs) purchased from Invitrogen were cultured in Medium 200 containing large vessel endothelial supplement at 37 °C in a humidified atmosphere of 5% CO_2_. Angiogenesis assay was performed according to the manufacturer’s instructions. Briefly, Geltrex LDEV-Free Reduced Growth Factor Basement Membrane Matrix (Invitrogen) was thawed at 4 °C overnight; the thawed Geltrex matrix solution was mixed by pipetting up and down, and 0.1 ml of Geltrex Matrix was added to each well of a 24-well plate (prechilled at −20 °C) and incubated for 30 min at 37 °C. The cells were then harvested and resuspended to 1.9 × 10^5^ cells/ml in unsupplemented medium, and 0.25 ml of the cell suspension was combined with 0.25 ml of cancer cell-conditioned medium and slowly added to a precoated well. At 17 h after incubation, the HUVECs were stained with a cell-permeable dye, calcein, and visualized under a ZEISS fluorescence microscope (×10, Carl Zeiss Microimaging GmbH, Germany).

### 2D electrophoresis and mass spectrometry analysis

2D electrophoresis (2DE) was performed by Kendrick Laboratories (Kendrick Labs, Inc.), and MALDI-TOF analysis was performed at the Protein Chemistry Core Facility (Columbia University). Briefly, cells were rinsed three times with cold PBS, and cellular lysates were prepared with an osmotic lysis buffer containing protease and phosphatase inhibitors, followed by a 3-min sonication. The lysates were then stored at −80 °C before shipping.

### Fluorescence in situ hybridization (FISH)

The expression of hsa-miR-34a in human breasts and other cancers (BRC961, BRC962, and MTU951 arrays; Pantomics) was determined by FISH, as detailed elsewhere [78]. Briefly, after deparaffinization, sections were prehybridized for 20 min at 55 °C, followed by a 1 h hybridization at the same temperature with a miRCURY LNA hsa-miR-34a detection probe (1:1000 dilution; Exiqon). After washing, the sections were blocked for 1 h with blocking solution and incubated with anti-digoxigenin-fluorescein Fab fragments (1:1000 dilution; Roche) at 4 °C overnight. The levels of tRNA_i_^Met^ were also detected in the same specimens by FISH analysis using a 5’-labeled, 2-F modified tRNA_i_^Met^ detection probe (GGT TTC GAT CCA TCG ACC TCT GGG TTA TGG GC, Creative Biolabs), as described previously [78], with some modifications. Briefly, the tissue sections were incubated with 400 μl of prehybridization buffer (4× SSC containing 50% deionized formamide) for 2 h at 37 °C in a humid chamber. The slides were immersed in 2× SSC for 5 min, and the tissue sections were then incubated with 400 μl of 20 μM probe diluted in hybridization buffer (10% dextran sulfate, 0.2% BSA, 2× SSC, 125 µg/ml tRNA, 500 µg/ml denatured sonicated salmon sperm DNA, and 1 U/ml RNasin) and covered with DEPC-treated coverslips at 37 °C overnight. After a sequential wash, the sections were incubated for 30 min with blocking solution (buffer 1 containing 0.1% Triton X-100 and 2% BSA), followed by a 2 h of incubation with a 1:1000 dilution of a sheep anti-DIG antibody (Roche) in blocking solution in a humid chamber. Using a shaking platform, the sections were washed for 20 min with PBS buffer prior to the dehydration and mounting steps. Fluorescence was observed under 400X using an inverted microscope (ZEISS). The stained tissue sections were analyzed independently by a pathologist and research scientists in a blinded manner.

Staining intensity was the criterion used for quantitating immunofluorescence staining. A range from 0 to 3 was used to classify the intensity: 0 = absence of staining; 1 = weak staining; 2 = moderate staining; and 3 = intense staining.

### Knockdown of tRiMetF31

MCF7 cells grown to 70% confluency were transfected with either 50 nM tRiMetF31 siRNA1 or 50 nM tRiMetF31 siRNA2 or 100 nM tRiMetF31 siRNA1 or 100 nM tRiMetF31 siRNA2 using Lipofectamine 3000 (Invitrogen) per the manufacturer’s instructions; the oligos were synthesized by Dharmacon; AllStars negative control siRNA was purchased from Qiagen. At 72 h after transfection, the medium was collected for tube-formation assay, and stored at -80 °C. The cells were washed twice with ice-cold PBS, lysed in a radioimmunoprecipitation assay buffer (RIPA), and stored at -20 °C. The sequences of tRiMetF31 siRNA duplexes used in this study are as follows: tRiMetF31 siRNA1 sense: 5’-AGA GUG GCG CAG CGG AAG CUU-3’, tRiMetF31 siRNA1 antisense: 5’-GCU UCC GCU GCG CCA CUC UUU-3’; tRiMetF31 siRNA2 sense: 5’-CAG AGU GGC GCA GCG GAA GUU-3’, tRiMetF31 siRNA2 antisense: 5’-CUU CCG CUG CGC CAC UCU GUU-3’.

### Bioinformatic analysis

The expression of tRNA_i_^Met^-derived fragments in breast cancer was analyzed using tRFexplorer, a public database developed previously [79]. Differential expression analysis was conducted using the DESeq2 Bioconductor package as part of functionality in tRFexplorer.

### Data extraction and meta-analysis

Two authors (BW and DL) individually determined inclusion/exclusion of all the retrieved articles, any discrepancy was resolved through discussion. Data extraction from each study was conducted independently by two authors (BW and DL). For each eligible article in which PFKFB3 expression was determined in breast cancer tissues by immunohistochemical staining, the following information was extracted: first author’s name, publication year, geographical populations, and the numbers of cases and controls.

RevMan5.4.1 software (Version 5.4.1, Cochrane 2020) was used for the current meta-analysis. The combined ORs and corresponding 95% CIs were calculated and computed in the forest plots for the PFKFB3 gene to evaluate the contribution of its expression to the risk of breast cancer, tumor size, and metastasis. A random-effect model was applied for the meta-analysis with the high heterogeneity (*I*^2^ > 50%); otherwise, a fixed-effect model was applied.

### Statistical analysis

Student’s *t* test was used for statistical significance of differences in miR-34a expression, tRiMetF31 expression, PFKFB3 expression, tRNA_i_^Met^ expression, luciferase activity, cell growth, apoptosis, cell cycle, cell migration, and angiogenesis between groups. *P* < 0.05 was considered significant.

## Supplementary information

Supplementary Figures, legends and table

Original Data

## Data Availability

All data needed to evaluate the conclusions of this paper are presented in the paper and/or Supplementary Materials. Additional data related to this paper may be requested from the authors.
