# Supplementary material for: A miR-34a-guided, tRNAiMet-derived, piR_019752-like fragment (tRiMetF31) suppresses migration and angiogenesis of breast cancer cells via targeting PFKFB3
Source: Cell Death Discov. 2022 Aug 12;8:355. doi: 10.1038/s41420-022-01054-w (PMC9374763; doi:10.1038/s41420-022-01054-w)
Supplement: Supplementary file 1 — Supplementary Figures, legends and table [file 41420_2022_1054_MOESM1_ESM.pdf]

Fig. S1. Identification of piRNAs highly homologous with tRiMetF31. (**A**) Homology between rno_piR_000194 and tRiMetF31. (**B**) Homology between hsa_piR_019752 and tRiMetF31.

Fig. S2. 2DE/MALDI TOF analysis of differentially expressed genes. Whole cellular lysates were prepared from stable breast cancer cell lines HCC1806/Dox(-), HCC1806/Dox(+), HCC1806/control shRNA, and HCC1806/$tRNA_i^{Met}$-shRNA. 2DE/MALDI TOF analysis was done by Kendrick Laboratories (Kendrick Labs, Inc.) and the Protein Chemistry Core Facility (Columbia University).

Fig. S3. Volcano plot of the significant relationship between log2-fold change and adjusted p-value. $TRNA_i^{Met}$ fragment ts-63, significantly downregulated in breast cancer, is shown in red, while upregulated is shown in blue. Log2FC = -1.594, adj. p-value = 8.58E-25.

A

```
> Query (01-31) matches with rno_piR_000194 (182749844-182749875)
 Length: 31 bases   E-value: 2.5e-06

Query:          01 AGCAGAGTGGCGCAGCGGAAGCGTGCTGGGC 31
                   |||||||||||||||||||||||||||||||
rno_piR_000194: 01 AGCAGAGTGGCGCAGCGGAAGCGTGCTGGGC 31
```

B

```
> Query (02-31) matches with hsa_piR_019752 (27745665-27745696)
 Length: 31 bases   E-value: 6.5e-06

Query:          02 GCAGAGTGGCGCAGCGGAAGCGTGCTGGGC 31
                   ||||||||||||||||||||||||||||||
hsa_piR_019752: 01 GCAGAGTGGCGCAGCGGAAGCGTGCTGGGC 30
```

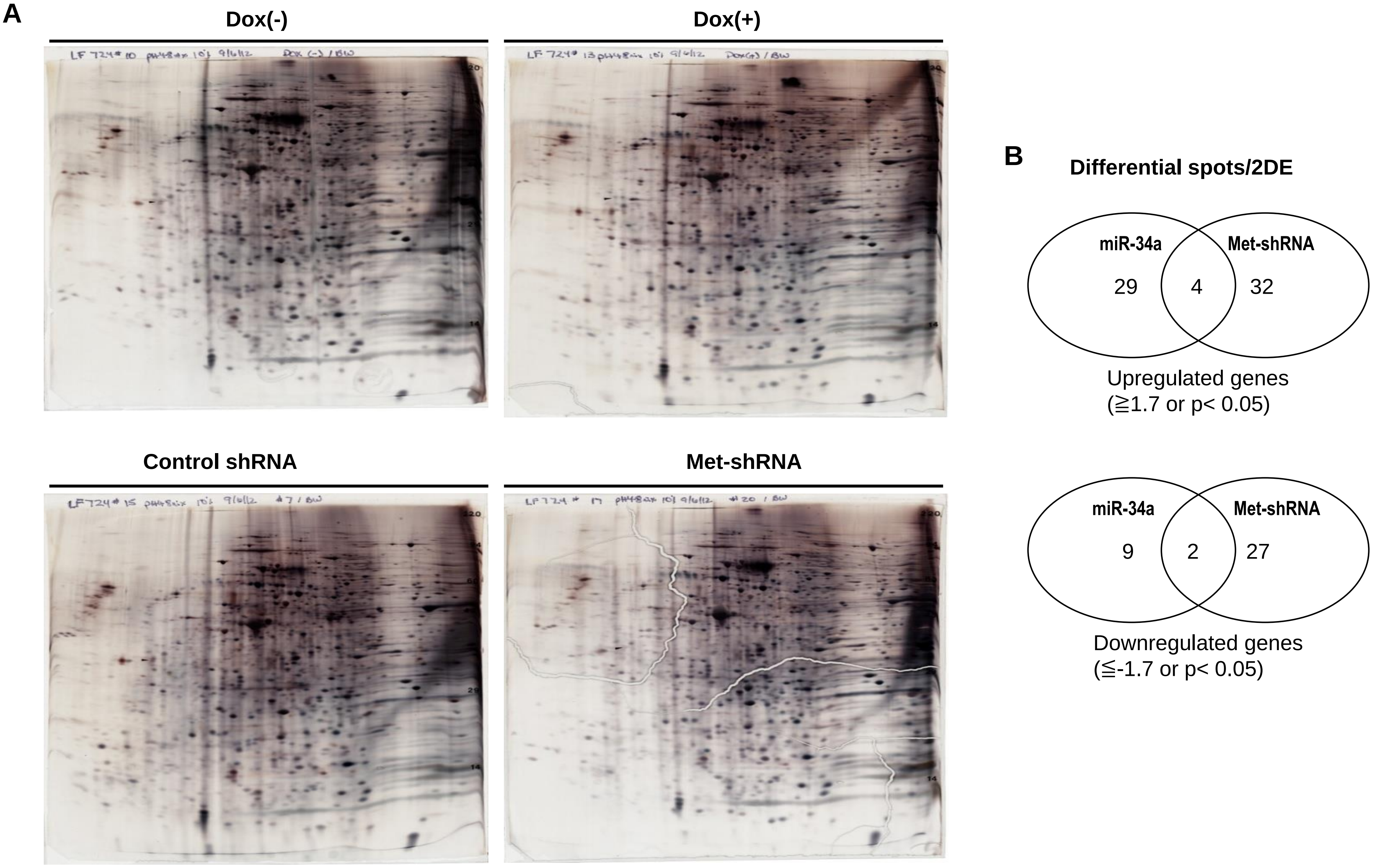
A
Dox(-)
Dox(+)
Control shRNA
Met-shRNA
B
Differential spots/2DE
miR-34a
Met-shRNA
29
4
32
Upregulated genes
(≧1.7 or p< 0.05)
miR-34a
Met-shRNA
9
2
27
Downregulated genes
(≦-1.7 or p< 0.05)

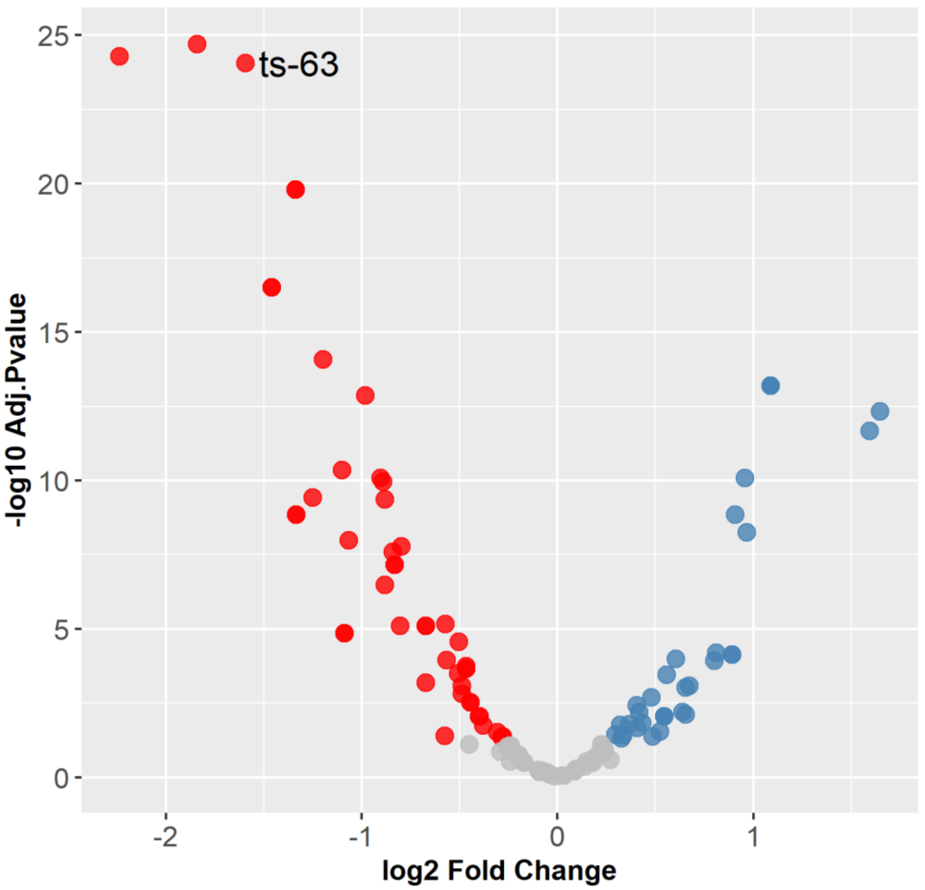

ts-63
25
20
15
10
5
0
-log10 Adj.Pvalue
-2
-1
0
1
log2 Fold Change

Table S1. Expression of $tRNA_i^{Met}$-derived fragments in breast cancer

**Fragment**	**Fragment type**	**Aminoacids**	**Anticodons**	**Expression cell lines**	**Expression in TCGA**
5P_tRNA-iMet-CAT-1-2	5' Leader	Met	CAT	MDA-MB-23; HS-578T	None in TCGA
**5P_tRNA-iMet-CAT-1-3**	**5' Leader**	**Met**	**CAT**	**MDA-MB-23; HS-578T;T-47D**	**Expressed in BRCA and other cancer types**
5P_tRNA-iMet-CAT-1-4	5' Leader	Met	CAT	MDA-MB-23	None in TCGA
5P_tRNA-iMet-CAT-1-8	5' Leader	Met	CAT	T47-D; MCF-7; MDA-MD-23	None in TCGA
5P_tRNA-Met-CAT-2-1	5' Leader	Met	CAT	T47-D; MDA-MB-23	None in TCGA
5P_tRNA-Met-CAT-3-2	5' Leader	Met	CAT	T47-D; MDA-MB-23	None in TCGA
5P_tRNA-Met-CAT-4-2	5' Leader	Met	CAT	MDA-MB-23; BT-549; HS-578T	None in TCGA
5P_tRNA-Met-CAT-7-1	5' Leader	Met	CAT	T47-D	None in TCGA
**ts-63**	**tsRNA**	**Met**	**CAT**	**T47-D; MCF-7; MDA-MD-23; BT-549; HS-578T**	**Expressed in BRCA and other cancer types**
