# Supplementary material for: A miR-34a-guided, tRNAiMet-derived, piR_019752-like fragment (tRiMetF31) suppresses migration and angiogenesis of breast cancer cells via targeting PFKFB3
Source: Cell Death Discov. 2022 Aug 12;8:355. doi: 10.1038/s41420-022-01054-w (PMC9374763; doi:10.1038/s41420-022-01054-w)

Original images: Figure 1D

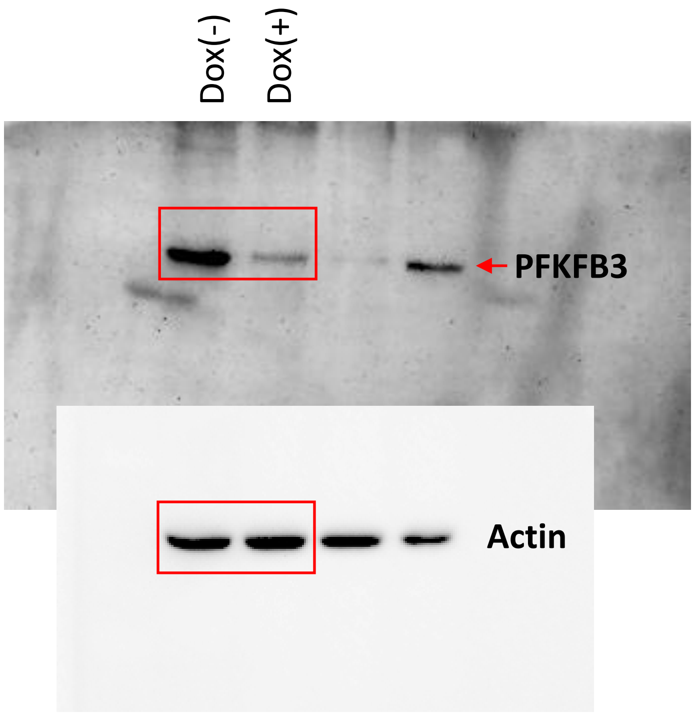

Original images: Figure 1G

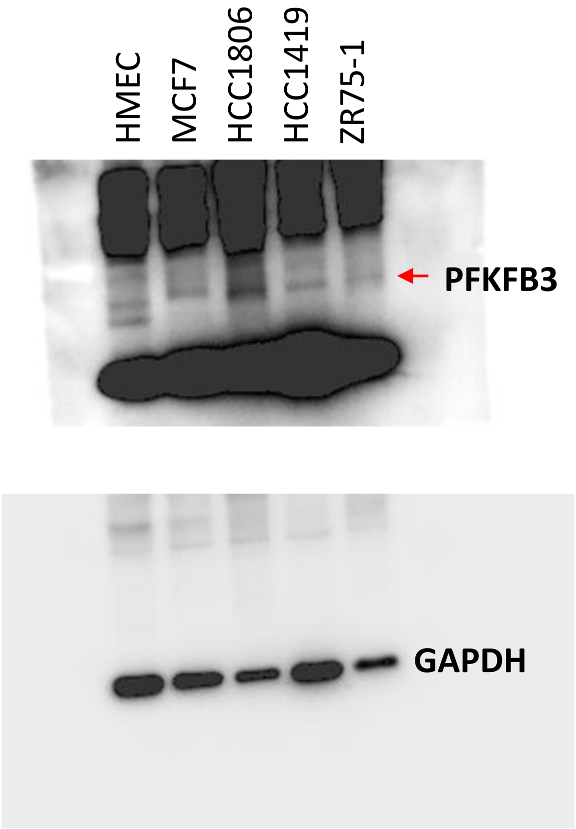

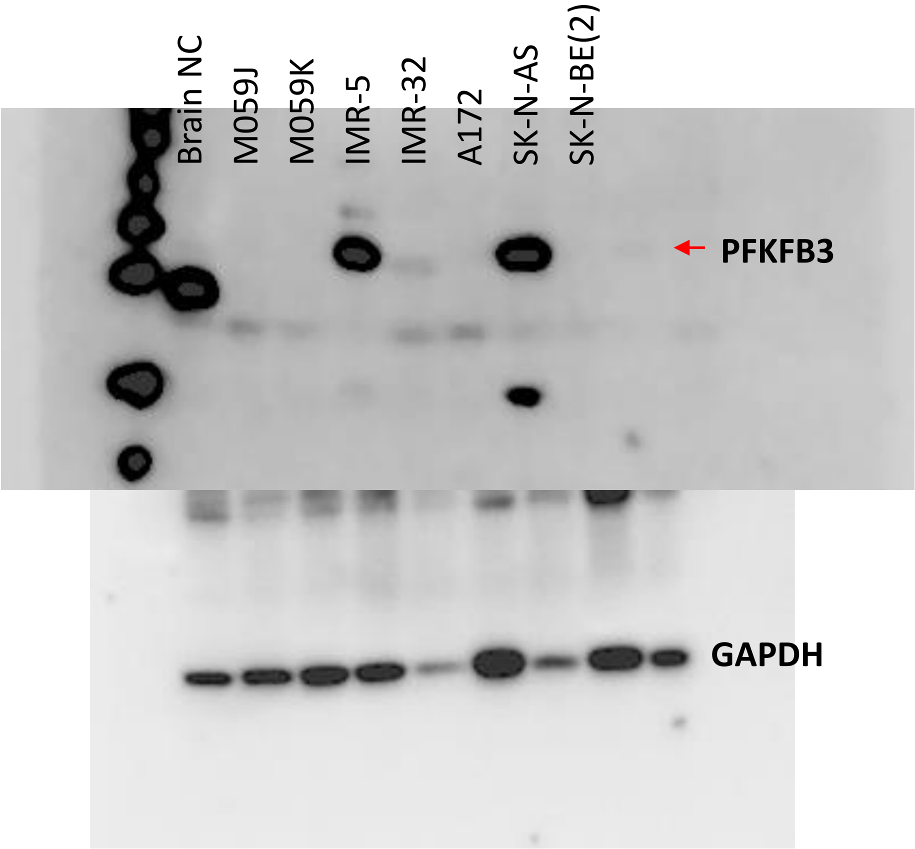

Original images: Figure 2A

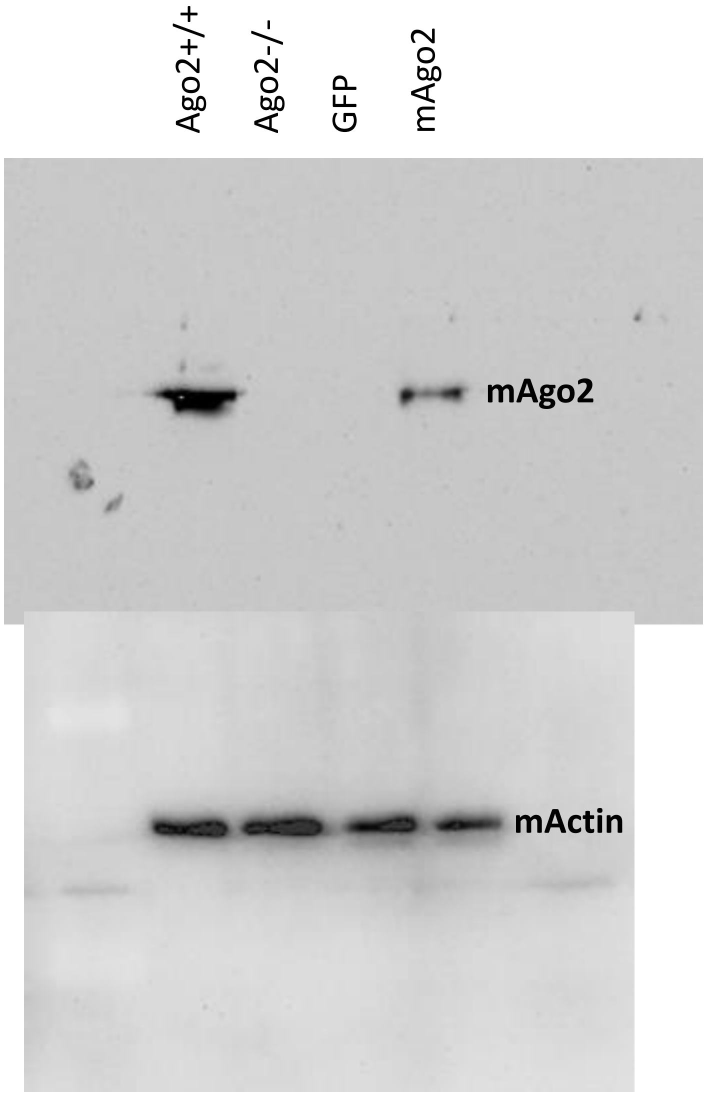

Original images: Figure 3A

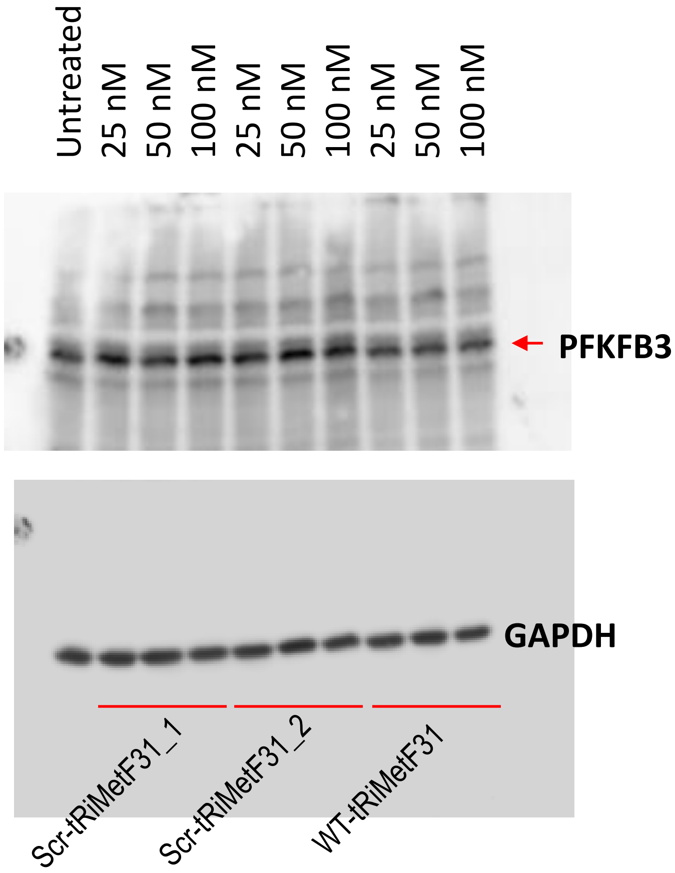

Original images: Figure 4A

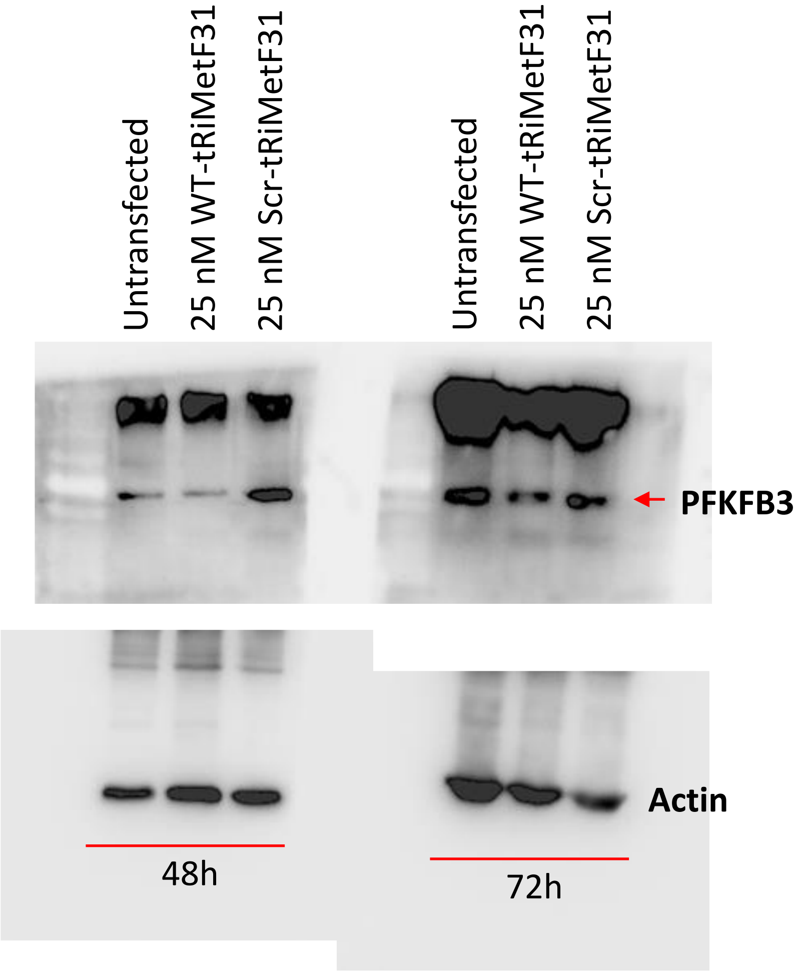

Original images: Figure 6F

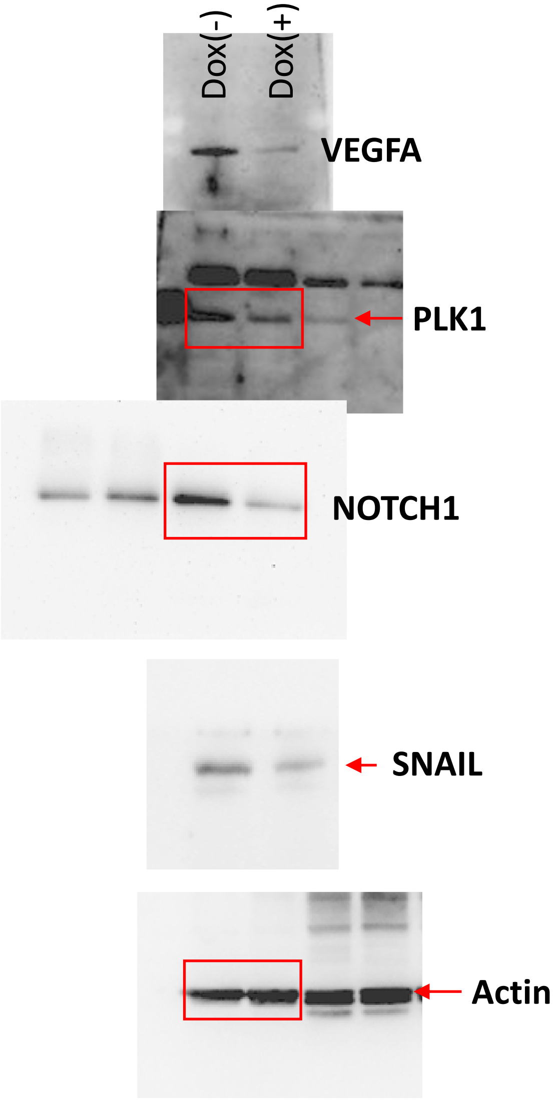

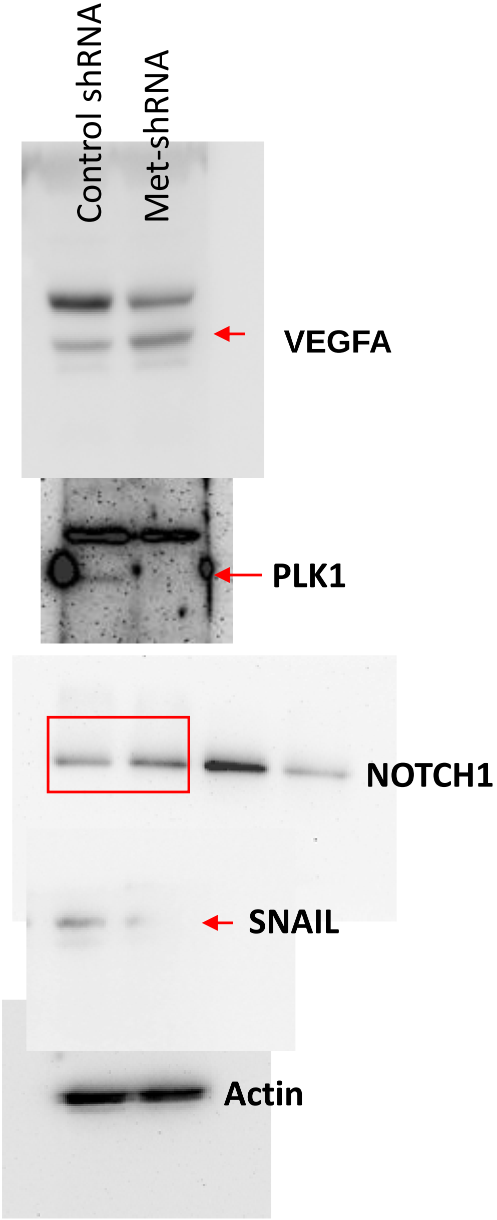

Original images: Figure 6G

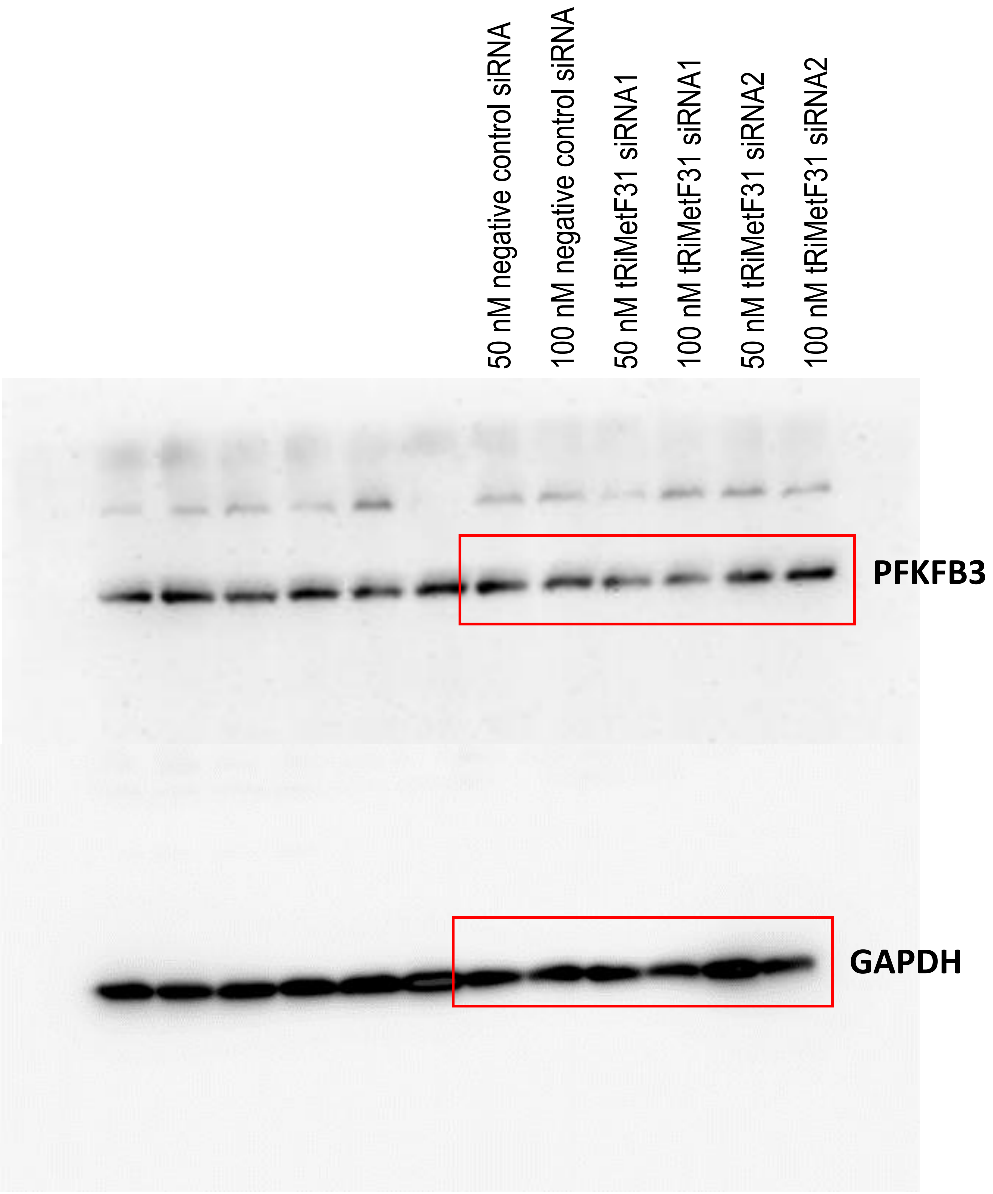

Supplement: Supplementary file 2 — Original Data [file 41420_2022_1054_MOESM2_ESM.pdf]
